# Towards a Four-Component GMMA-Based Vaccine against *Shigella*

**DOI:** 10.3390/vaccines10020328

**Published:** 2022-02-18

**Authors:** Francesca Micoli, Usman N. Nakakana, Francesco Berlanda Scorza

**Affiliations:** GSK Vaccines Institute for Global Health (GVGH) S.r.l., 53100 Siena, Italy; usman.n.nakakana@gsk.com (U.N.N.); francesco.x.berlandascorza@gsk.com (F.B.S.)

**Keywords:** *Shigella*, GMMA, outer membrane vesicles, OMV, vaccine

## Abstract

Shigellosis remains a major public health problem around the world; it is one of the leading causes of diarrhoeal disease in low- and middle-income countries, particularly in young children. The increasing reports of *Shigella* cases associated with anti-microbial resistance are an additional element of concern. Currently, there are no licensed vaccines widely available against *Shigella*, but several vaccine candidates are in development. It has been demonstrated that the incidence of disease decreases following a prior *Shigella* infection and that serum and mucosal antibody responses are predominantly directed against the serotype-specific *Shigella* O-antigen portion of lipopolysaccharide membrane molecules. Many *Shigella* vaccine candidates are indeed O-antigen-based. Here we present the journey towards the development of a potential low-cost four-component *Shigella* vaccine, eliciting broad protection against the most prevalent *Shigella* serotypes, that makes use of the GMMA (Generalized Modules for Membrane Antigens) technology, a novel platform based on bacterial outer membranes for delivery of the O-antigen to the immune system.

## 1. Introduction

*Shigella* spp. are a leading cause of diarrhoeal mortality among all ages worldwide [1,2,3]. The spectrum of disease ranges from mild diarrhoea to severe dysentery (bloody, mucoid diarrhoea) with fever. Recent estimates report approximately 270 million diarrhoea episodes due to *Shigella,* with around 212,000 deaths among all ages per year, of which 64,000 are in children under 5 years. Among these deaths, 90% occur in low- and-middle-income countries (LMICs) [1]. Although mortality rates from diarrhoeal diseases have decreased since 1990, diarrhoea morbidity remains high, particularly in LMICs, where access to health care, relevant microbiological diagnostics, quality of water, and sanitation are poor, and there is poor access to adequate health-care facilities, diagnostics, and therapeutic interventions. Complications from infection are especially common in malnourished children, with possible long-term consequences including stunting and cognitive impairment in children experiencing multiple diarrhoeal episodes [4]. Additionally, diarrhoea due to *Shigella* can cause significant morbidity among travellers and military recruits. Antibiotic resistance of *Shigella* is increasing, and conventional therapeutic antibiotics against shigellosis have become progressively less efficient. Many outbreaks of shigellosis have been reported due to resistant strains [5,6], and the WHO’s Global Antimicrobial Resistance Surveillance System has identified *Shigella* as a priority pathogen for the development of new interventions [7].

There are four different *Shigella* species—*S. dysenteriae*, *S. flexneri*, *S. boydii*, *S. sonnei*—divided into more than 50 different serotypes based on the outer polysaccharide antigen (O-antigen, OAg) of the lipopolysaccharide (LPS) expressed on the bacteria surface [8]. Challenge and rechallenge studies in nonhuman primates [9] and volunteers [10,11,12], epidemiological field studies [13], and seroepidemiological studies [14,15] have suggested that clinical infection with wild-type *Shigella* strains confers approximately 75% serotype-specific immunity [3]. Live oral vaccines [16,17,18,19] and O-polysaccharide-protein conjugate vaccines [20,21] that have conferred protection in randomized controlled field trials have corroborated the importance of immune responses to *Shigella* OAg. The prevalence of *Shigella* serotypes varies by country economic status and between geographical regions and changes over time even within one region. These changes present a major challenge for vaccine development. Incidence data from specific sites of the Global Enteric Multicentre Study (GEMS) in sub-Saharan Africa and South Asia show that 89.6% of *Shigella* case isolates were *S. flexneri* (65.9%) or *S. sonnei* (23.7%) and confirm *Shigella* infection among the top causes of potentially life-threatening diarrhoeal illness among infants and children [2,22].

Currently, there are no licensed vaccines widely available for *Shigella*, but several candidates are being evaluated at different stages of preclinical and clinical development [23,24]. Human and animal challenge trials with virulent *Shigella*, as well as observational studies in endemic areas, showing that the incidence of disease decreases following a prior *Shigella* infection, support the feasibility of a vaccine [25]. Serum and mucosal antibody responses are predominantly directed against the serotype-specific *Shigella* OAg, and protection from subsequent infection has been correlated with increased levels of these antibodies [13,14,26,27,28]. Consequently, many *Shigella* vaccine candidates are indeed OAg-based. Numerous strategies, including live attenuated oral, killed oral, and subunit parenteral vaccines, are actively being explored. If the cross protection observed in animals can be extrapolated to humans, multivalent vaccines against *S. sonnei* and the most common circulating *S. flexneri* serotypes are expected to achieve approximately 65% coverage, which could further increase to over 85% depending upon the degree of cross-protection elicited against *S. flexneri* strains not contained in the vaccine [3]. Finally, new vaccine candidates are being explored to leverage conserved protein antigens in addition to or instead of OAg [24].

Here we present the journey towards the development of a potential low-cost four-component *Shigella* vaccine, eliciting broad protection against the most prevalent *Shigella* serotypes, that makes use of the GMMA (Generalized Modules for Membrane Antigens) technology, a novel platform based on bacterial outer membranes for delivery of the OAg to the immune system [29,30].

## 2. Generalized Modules for Membrane Antigens Platform

Gram-negative bacteria (e.g., *Shigella*) have an outer membrane and an inner membrane linked through multiple protein systems. They naturally shed small amounts of outer membrane vesicles (OMV) by a process of blebbing. Indeed, spontaneous release of vesicles from bacteria results in the formation of native OMV, but for many species this occurs at very low levels, insufficient to be purified for inclusion in a vaccine formulation. To overcome low yields, Gram-negative bacteria can be genetically manipulated to greatly increase the shedding of outer membranes [31].

A reverse vaccinology study on *N. meningitidis* identified genes yielding a hyper-blebbing phenotype and first suggested a possible application of OMV as a platform for vaccine development [32,33]. While the genes identified in the *N. meningitidis* reverse vaccinology study were specific to *N. meningitidis*, a forward genetic approach based on genome-wide transposon screening in *E. coli* laboratory strain identified multiple null mutations that generate a similar hyper-blebbing phenotype. This included disruption of the Tol-Pal system, which is highly conserved across Gram-negative bacteria [34]. In a subsequent study, a *tolR* null mutation was induced in extraintestinal *E. coli* (ExPEC) pathogenic strains and first demonstrated immunogenicity and protection conferred by OMV generated with this technology [35]. Following studies expanded this approach as a generic platform for homologous and heterologous protein expression system for vaccine applications [36].

*Shigella sonnei* was engineered to overexpress OMV via *tolR* null mutation, and additional mutations were added to minimize the capacity of GMMA to promote a reactogenic response when delivered in a parenteral formulation [31]. This approach allowed reactogenicity to be efficiently reduced, while maintaining the immunopotentiator effect of the Toll-like receptor 4 (TLR4) triggered by lipid A [37,38,39]. Additional mutations were developed to remove antigens detrimental to a vaccine or to enhance safety (e.g., Δ*virG*) [40].

GMMA nomenclature was introduced to distinguish vesicles naturally released from genetically mutated bacteria from OMV produced by detergent extraction from wild-type bacteria (dOMV). Almost all GMMA protein is outer membrane or periplasmic, with low contamination by cytoplasmic proteins [31,41,42]. GMMA have been generated from multiple Gram-negative bacteria including: *S. sonnei* [40,42]; *S. flexneri* serotypes [43,44,45]; *Salmonella enterica* serovars Typhimurium, Enteritidis, and Paratyphi A [46,47,48,49]; *Neisseria meningitidis* serogroup A and W strains [50].

GMMA can be produced at high yields using a simple and robust process of manufacturing, potentially leading to low-cost vaccines [51]. In fact, after fermentation of the mutated bacteria, GMMA are purified with a rapid filtration-based downstream processing. GMMA can be produced at high yields using simple, robust, and cost-effective manufacturing processes and were quickly recognized as an ideal technology for low resource settings, where the ability to produce and deliver vaccines sustainably in markets without strong commercial drivers is critically important and where poverty-related conditions such as enteric and diarrhoeal diseases are among the leading causes of mortality in children.

Importantly, GMMA mimic the outer membrane of bacteria, where key antigenic components to elicit an immune response are lacking the ability to cause associated disease. They contain pathogen-associated molecular patterns (e.g., lipopolysaccharides, lipoproteins) conferring self-adjuvanticity and have an optimal size for immune stimulation, with potential for strong immunogenicity [30].

On the other hand, GMMA are quite complex, and a comprehensive panel of analytical methods has been put in place to characterize GMMA as particles and for analysis of key antigens displayed on their surface to ensure quality and consistency of manufacture at release and to follow GMMA stability over time [52,53,54].

## 3. *Shigella* Generalized Modules for Membrane Antigens

### 3.1. Preclinical Experience

The first *S. sonnei* strain was genetically modified to increase blebbing (Δ*tolR*), produce penta-acylated lipid A (Δ*htrB*) with reduced endotoxicity, and stably express the virulence plasmid encoding for the immunodominant OAg component of the LPS (by replacing the plasmid-encoded *virG* gene with *E. coli nadA* and *nadB* genes to remove the nicotinic acid auxotrophy) [40]. The scale-up of the process, consisting of two simple tangential flow filtration steps after fermentation of the mutated bacteria [31], to GMP pilot scale yielded a very robust process that generates approximately 100 mg of GMMA proteins per litre of fermentation, meeting predetermined product specifications.

As GMMA contain LPS and lipoproteins, assessing potential reactogenicity was prioritized for vaccine development. In an in vitro monocyte activation test (MAT), *S. sonnei* GMMA showed a markedly lower IL-6 production compared with peripheral blood mononuclear cells (PBMC) stimulated with the parent GMMA with unmodified lipid A [40]. It was found that the residual activity of GMMA was largely due to non-lipid A-related TLR2 activation [39]. Additionally, a modified rabbit pyrogenicity test was established, based on the European Pharmacopoeia pyrogens method, but using intramuscular administration of the full human dose (100 μg of protein) of *S. sonnei* GMMA adsorbed on Alhydrogel (1790-GAHB) [40].

Aluminium hydroxide was used as adsorbent to further reduce potential reactogenicity of GMMA particles [55]. Minor temperature increases were observed in rabbits receiving the vaccine, but these were transient and within an acceptable range. A repeat dose toxicology study in rabbits using intramuscular (IM, 6.4/100 μg/dose), intranasal (IN, 5.1/80 μg/dose), or intradermal (ID, 0.64/10 μg/dose) administration routes showed good tolerability of the vaccine by all routes, supporting its suitability for use in humans [40]. As for the rabbit toxicology studies, the Irwin test in rats showed no evidence that IN immunization with 1790GAHB could cause neurological findings. In mice, 1790-GMMA formulated on Alhydrogel elicited anti-LPS specific serum IgG in a dose-dependent manner. Vaccination of mice with 1.75 ng of OAg/29 ng protein gave an already significant anti-LPS IgG response after a single injection, with a strong increase following a second injection 4 weeks later [40]. At the doses tested in the rabbit toxicology study, the antibody response peaked after the first dose IM, while after the second dose IN and ID.

**Figure 1:**
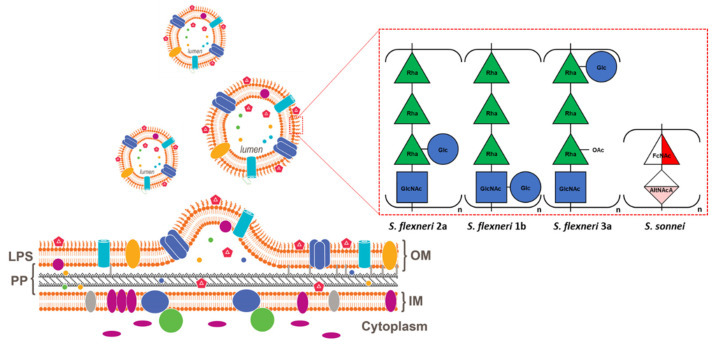
Schematic representation of GMMA formation and combination of *S. sonnei*, *S. flexneri* 1b, 2a and 3a GMMA in the four-component OAg-based *Shigella* vaccine candidate altSonflex1-2-3. LPS: lipopolysaccharide; PP: periplasm; IM: inner membrane; OM: outer membrane; Rha: Rhamnose; Glc: Glucose; GlNAc: N-acetyl glucosamine; FcNAc: 2-N-acetyl 4-fucosamine; AltNAcA: 2-N-acetyl alturonic acid; OAc: O-acetyl.

Strategies applied to *S. sonnei* strain for GMMA production were demonstrated also for *S. flexneri* strains [39,56]. *S. flexneri* serotypes, in contrast with *S. sonnei* and with the exception of *S. flexneri* 6, share a common OAg backbone. To assess the potential for immunologic cross-reactivity of *S. flexneri* GMMA, 14 different *S. flexneri* GMMA producer strains (Δ*tolR*) were generated [56]. Corresponding GMMA were tested in mice for their ability to induce cross-reactive and cross-functional antibodies. GMMA, eliciting antibodies cross-binding (by FACS) and cross-killing (by serum bactericidal assay, SBA) on numerous heterologous *S. flexneri* serotypes were identified, suggesting that broadly protective *Shigella* vaccines are possible and may cover multiple serotypes with a limited number of components. For example, *S. flexneri* 1b GMMA elicited antibodies able to bind and kill different heterologous serotypes, including *S. flexneri* serotypes 4a and 6. On the other hand, narrow specificity GMMA immunogens were identified, such as *S. flexneri* 2a, inducing antibodies able to bind to and kill only the homologous serotype bacteria or few others. These data informed the design of a four-component vaccine based on GMMA from *S. sonnei*, *S. flexneri* 1b, *S. flexneri* 2a, and *S. flexneri* 3a (Figure 1). The resulting formulation tested in mice and rabbits elicited strong SBA titres against a large panel of *Shigella* serotypes [56].

This study also highlighted the importance of anti-OAg antibodies for binding and inducing bactericidal activity against OAg-positive bacteria. Actually, antibodies generated against OAg-negative GMMA were unable to bind to OAg-positive bacteria. These results agreed with the recent work of Mancini et al. [42], demonstrating that anti-protein antibodies are induced in mice upon immunization with either OAg-negative or OAg-positive *S. sonnei* GMMA, but the presence of OAg chains on the bacterial surface shield the bacteria from anti-protein antibody binding, and therefore anti-OAg antibodies are the main drivers of bactericidal activity against OAg-positive bacteria. This finding is reinforced by the functional analysis of human sera from a phase 2b study of the *S. sonnei* GMMA-based vaccine [57]. The adsorption of anti-OAg antibodies from post-vaccination sera confirmed that anti-protein antibodies were not able to induce complement-mediated bactericidal killing against *S. sonnei* OAg-positive bacteria.

Interestingly, antibodies that are not targeting the Oag are functional against Oag-negative bacteria, and immunodominant protein antigens were identified by proteomic analysis [42]. Little is known about OAg length and density regulation during infection and, therefore, protein exposure. It is possible that protein antigens could contribute to protection through other mechanisms [58]. Ongoing activities are focused on understanding the role of proteins in protection and whether the presence of protein antigens on *Shigella* GMMA represent an added value for GMMA vaccines compared with other OAg-based formulations. It has already been demonstrated that GMMA-associated proteins stimulate cooperation of B-cells with T-helper cells for induction of a T-cell dependent anti-OAg specific response. In recently published works, GMMA were shown to induce a stronger response in wild-type than in T-cell-deficient mice, clearly highlighting a strong T-dependent component of the immune response induced by GMMA [43,44]. Interestingly, when presented on GMMA, even short OAg chains are able to elicit part of their response through direct activation of B-cells without involving T-cells. This could be explained by the particulate nature of GMMA displaying highly organized and repetitive antigens (i.e., OAg) to the immune system. Such multiplicity can favour efficient B-cell receptor (BCR) crosslinking, delivering strong activation signals to the B-cells independent of polysaccharide length [59]. Moreover, GMMA contain different TLR agonists, including lipid A and lipoproteins, able to provide synergistic TLR signalling to B-cells [37], as confirmed by MAT. This could favour a rapid and strong response after one only injection [60]. Even if a purer T-dependent response could have a positive impact on memory and persistency of the antibody response, when compared head to head in a mouse study, no differences were observed in the ability of GMMA or traditional glycoconjugates to induce strong responses after re-injection or in the persistence of the antibody response induced [43]. In this study, GMMA induced higher anti-O-antigen IgG than glycoconjugate administered without Alhydrogel, as already verified with *Salmonella* GMMA [49]. Additional studies are currently ongoing to compare four-component GMMA and glycoconjugate formulations in animal models.

A series of studies was performed to better assess the criticality of *Shigella* GMMA attributes. The expertise acquired on bacteria manipulation allowed the efficient generation of strains characterized by different OAg length or different O-acetylation pattern. *Shigella* OAg biosynthesis is dependent on the Wzx/Wzy pathway, and the degree of repeating unit polymerization (i.e., OAg size) is regulated by the Wzz family of proteins, responsible for unique polysaccharide modal lengths [61]. OAg repeating units can also be assembled into high molecular weight capsules (Group 4 Capsules, G4C) if an additional G4C operon is present, as it is in the case of *S. sonnei* and *S. flexneri* 6 serotypes [43,44,62]. Consequently, OAg populations at different length are displayed on GMMA. Mutations were introduced in the OAg locus or in the G4C operon in order to display homogeneous polysaccharide populations of different sizes on GMMA surface. For *S. sonnei*, *S. flexneri* 2a, and *S. flexneri* 6, characterized by OAg with different structural characteristics (zwitterionic, neutral, and negatively charged sugars), no major impact of OAg chain length on the immune response elicited in mice was verified [43,44].

GMMA were also engineered to express OAg with different O-acetylation patterns [45]. The role that O-acetylation can have on the immune response elicited by *S. flexneri* OAg can be important not only to assess the protective role against homologous strains, but also against heterologous serotypes sharing common epitopes due to such modifications. *S. flexneri* 2a and 1b OAg with different O-acetylation patterns were displayed on GMMA by knocking-out the responsible O-acetyltransferase genes. The data obtained suggested that the OAg O-acetylation is not critical for the generation of functional antibodies against homologous or heterologous strains.

### 3.2. Clinical Experience with S. sonnei Monocomponent GMMA Vaccine

*S. sonnei* GMMA formulated on Alhydrogel (1790GAHB) has been tested in five clinical trials to date (Table 1).

Two parallel phase 1 trials in European adult population (18–45 years) were performed [63]. During the two phase 1 trials, a range of doses from 1.5/25 µg (OAg/protein) to 6/100 µg were tested and different routes using IM, ID, and IN routes. The maximum dose of OAg was limited by the amount of protein that could be administered without compromising on participant safety.

The initial phase 1 study (H03_01TP, clinicaltrial.gov NCT02017899) evaluated the safety and immunogenicity profile of three IM injections at 4 weekly intervals compared with placebo. *S. sonnei* 1790-GMMA containing 12 μg/mL of OAg and 200 μg/mL protein (6/100 μg dose) was adsorbed to 0.7 mg Al^3+^/mL of aluminum hydroxide. Dilutions of the full dose with placebo were performed to obtain the other doses tested. Intramuscular doses ranged from 0.06/1 µg to 6/100 µg (OAg/protein content) in an injected volume of 0.5 mL. In the second phase 1 study (H03_02TP, clinicaltrial.gov NCT02034500), the safety and immunogenicity of 1790GAHB was also evaluated when delivered three times, 4 weeks apart at different doses intradermally or intranasally, testing other routes of immunization which have different theoretical advantages, in addition to a cohort including IM administration. Intradermal doses were from 0.006/0.1 μg to 0.6/10 μg in 0.05 mL and IN doses were 0.3/5 μg to 4.8/80 µg in a total volume of 0.4 mL administered as a 0.2 mL aliquot into each nostril.

In both studies, vaccines were well-tolerated after administration by IM, ID, and IN vaccination routes. Solicited events were mild to moderate in severity. Vaccine-related unsolicited adverse events were uncommon. No subjects experienced serious adverse events (SAEs). Among unsolicited adverse events, eight cases of neutropenia were reported in vaccinees [64]. All cases of neutropenia were transient and had a benign clinical outcome. In the phase 1 and subsequent trials, no pyrogenic response was observed post-vaccination. In line with the outcome from preclinical rabbit studies [40], in the phase 1 common solicited local adverse event was limited and primarily confined to injection-site pain. Rhinorrhoea was the most common adverse event by the IN route. A correlation between dose and local reactions was observed, but even at the highest dose of 6/100 µg, the vaccine had an acceptable reactogenicity profile.

When delivered by the IM route, by pooling together the responses to the 1.5/25, 3/50, and 6/100 μg doses (defined as high-dose groups), reverse cumulative antibody distribution 28 days after the second vaccination (day 85) for the high-dose group showed a similar distribution to the antibody responses measured in an adult population following natural infection, with a median of 305 ELISA units compared with 121 ELISA units for the convalescent patients and a substantial increase compared with the pre-vaccination distribution [27,63]. On the last day of the study (day 225), the median antibody in vaccinees was 241 ELISA units, higher than the median in the convalescent patients. Moreover, antibodies with complement-mediated bactericidal activity were detected in vaccinees but not in placebo recipients, and correlation between SBA titres and anti-*S. sonnei* LPS serum IgG levels was observed [65].

Despite screening prior to enrolment, about half of the subjects had detectable anti-*S. sonnei* LPS antibodies prior to vaccination. Individual antibody responses suggested qualitative and quantitative differences between subjects with and without preexisting detectable antibody. Subjects with preexisting antibody tended to have stronger responses to the vaccine and a different pattern of boosting and decay kinetics.

In contrast with preclinical animal results [40], via the ID or IN routes, the vaccine was poorly immunogenic at the doses tested. The finding of no significant response following intranasal administration is consistent with findings following the administration of another *Shigella* vaccine [66]. It may be that the doses administered in our trial were too low to elicit an immune response. Additional studies could be useful for exploring higher ID and IN doses in an attempt to identify the optimal immune response and to expand the use of GMMA to mucosal vaccination.

Subsequently, an extension of the initial IM phase 1 study evaluated memory response induced by booster vaccination with 1.5/25 µg of 1790GAHB in individuals who had undetectable antibodies at baseline in H03_01TP. Prior to vaccination, specific antibody levels in some subjects immunized 2–3 years earlier were well-maintained, suggesting good immune persistence from vaccination. 1790GAHB was confirmed to be well-tolerated and to induce a significant booster response in previously primed adults, even those receiving primary doses of OAg/protein content as low as 0.059/1 µg, both in terms of anti-LPS IgG response and bactericidal activity, regardless of the priming dose. Additionally, the proportion of subjects achieving convalescent antibody levels (121 EU) 7 days post booster was 71% compared with 24% in the naïve group, suggesting a good immunological memory [65,67].

Based on the results from the two phase 1 trials, the IM route was selected for further development in a phase 2a trial (H03_04TP, clinicaltrial.gov NCT02676895) in African adults aged 18–45 years. Participants received two vaccinations with the 1790GAHB vaccine at doses of either 1.5/25 μg of OAg/protein or 6/100 μg 1 month apart, or vaccination with active comparator vaccines (a quadricomponent meningococcal vaccine at day 1 and tetanus, diphtheria, and acellular pertussis vaccine at day 29). The most frequently reported solicited local and systemic adverse events across all groups were pain and headache, respectively, with a similar reporting frequency between participants receiving the 1790GAHB and active comparators. One case of severe systemic reaction was reported (severe headache after first vaccination in group 6/100 μg). Neutropenia was designated as an adverse event of special interest (AESI) to allow systematic data collection for those events, based on the cases observed in the previous studies. Ten episodes of neutropenia were reported in two (9%), three (12%), and one (4%) participants in the 1.5/25 µg, 6/100 µg, and active comparator groups, respectively. These episodes were all considered probably or possibly related to vaccination; two of them occurred in the 1.5/25 µg group (one mild and one moderate), five in the 6/100 µg group (four mild and one moderate), and three in the control group (one mild, one moderate, and one severe). All cases of neutropenia were transient (recovering within 7 days and by study end) and asymptomatic, as confirmed by daily home visits during the 7 days post vaccination. No SAEs were reported [68].

Anti-*S. sonnei* LPS serum IgG geometric mean concentrations were evaluated at days 1, 29, and 57 and compared with anti-*S. sonnei* LPS antibody levels in convalescent patients naturally exposed to *S. sonnei*. Very high baseline anti-*S. sonnei* LPS serum IgG levels were observed in the study population; the median antibody levels were 30-fold higher than observed in Israeli convalescent sera and in healthy European adults vaccinated with the same doses of 1790GAHB. Despite this, the 1790GAHB vaccine induced robust antibody responses in Kenyan adults. At day 29, geometric mean antibody levels increased 2.10-fold and 4.43-fold from baseline in volunteers immunized with 1.5/25 μg and 6/100 μg of 1790GAHB, respectively. Day 57 antibody levels in the 1790GAHB-immunized groups were not statistically different from those at day 29. No increase was observed among controls. In the 1.5/25 μg dose group, seroresponse was 68% after first and 90% after second immunization; a 96% seroresponse was observed regardless of immunization in the volunteers receiving 6/100 μg.

**Figure 2:**
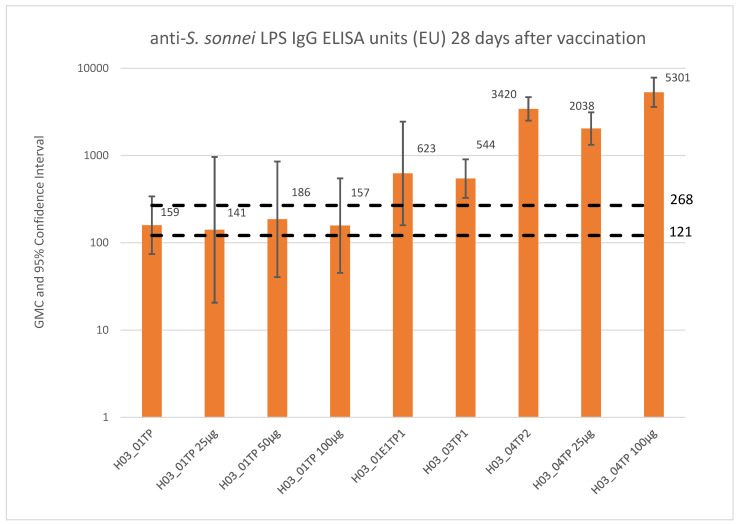
Summary of anti-LPS IgG response elicited by *S. sonnei* GMMA after IM immunization of European adults after primary vaccination (H03_01TP) or booster (H03_01E1TP), adults in the US (H03_03TP), and of adults in a disease-endemic area (H03_04TP). The 121 EU dashed line represents the level of antibodies in an adult population following natural infection measured with the ELISA assay used for H03_01TP, H03_04TP, and H03_01E1TP trials, while the 268 EU dashed line is the equivalent for H03_03TP. GMCs were calculated from all subjects with available data.

The 1790GAHB studies described above provided evidence that the vaccine was well tolerated and immunogenic in both European and African adults (Figure 2) and in the presence of preexisting antibody, vaccine- or infection-induced.

To evaluate the efficacy of the vaccine in a challenge human infection model (CHIM) for shigellosis, a phase 2b trial was performed. Additional objectives were to evaluate safety and immunogenicity of 1790GAHB in healthy US adults aged 18–45 (H03_03TP, clinicaltrial.gov NCT02676895). Two IM injections with 1.5/25 µg (OAg/protein) of 1790GAHB, administered one month apart, or a placebo control were followed by an oral challenge of volunteers with live *S. sonnei* 53G, 4 weeks after the second vaccination. Although safe and immunogenic, under the trial conditions, the vaccine did not show any efficacy against shigellosis [57]. Regarding neutropenia, following the evaluation of the signal of asymptomatic neutropenia among mostly participants of African descent in the phase 1 and phase 2a studies, and the phase 2b study, which showed that the *S. sonnei* 1790GAHB was not more likely to cause neutropenia than other licensed vaccines, it was thus no longer considered as an AESI for further *Shigella* GMMA vaccine development.

**Figure 3:**
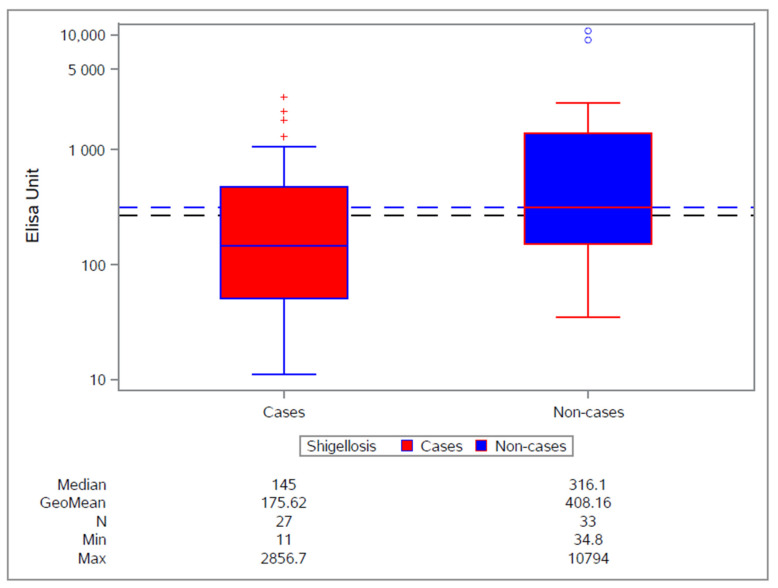
H03_03TP study: anti-*S. sonnei* LPS serum IgG by shigellosis at pre-challenge visit (overall population). +: Refers to individual subject antibody titres for the cases. o Refers to individual subject antibody titres for the non-cases.

Evidence was shown from the challenge trial; however, the evidence was in agreement with data previously published [26], showing that anti-LPS IgG antibody plays a significant role in protection against shigellosis (i.e., anti-*S. sonnei* LPS serum IgG concentration was higher among participants who did not develop shigellosis compared with participants who developed the disease following the challenge dose) (Figure 3). However, insufficient immunogenicity was considered to be the main root cause for the lack of efficacy. This finding suggested the need to enhance *S. sonnei* immunogenicity by increasing the OAg dose as well as by increasing the likely effect of the second vaccination, which in this trial showed no further increase in antibody levels from the levels after the first vaccination. This can be done by increasing the interval between the first and second vaccinations.

## 4. Moving toward a Four-Component *Shigella* GMMA-Based Vaccine

An alternative *S. sonnei* construct and *S. flexneri* GMMA have been developed to have higher OAg density on GMMA particle compared with the original *S. sonnei* GMMA, for inclusion in a four-component *Shigella* formulation. This will allow an increase in the dose of each OAg with no impact on safety, maintaining the total dose of proteins and lipid A below the highest dose tested in clinical trials with acceptable safety in 1790GAHB. The appropriateness of increasing the OAg dosage, with respect to the 1.5 μg OAg tested in the challenge trial with 1790GAHB, is also supported by observations carried out in previous clinical trials performed with the 1790GAHB vaccine, which showed an immunogenicity dose response [65,68], and by the much higher antigen content used for other OAg-based *Shigella* vaccines. The Flexyn2a vaccine, produced by Limmatech biotechnology in vivo in *E. coli* and containing 10 µg of *Shigella flexneri* 2a OAg was tested in a phase 2b proof of concept CHIM trial [69]. The Flexyn2a vaccine administered twice, one month apart intramuscularly, was found to be safe and immunogenic and demonstrated a protective efficacy point estimate of 72.4% against severe diarrhoea post-challenge. Another OAg-based candidate vaccine, the SF2a-TT15, containing 10 μg oligosaccharide conjugated to tetanus toxoid was tested in healthy adults in Israel. The vaccine was also shown to be more immunogenic at the 10 µg dose compared with the 2 µg dose [70]. Finally, the US NIH *S. sonnei* conjugate vaccine demonstrating 74% protective efficacy against shigellosis in adults [20] contained 25 µg of OAg conjugated to *Pseudomonas aeruginosa* recombinant exoprotein A (*r*EPA).

A clinical trial in European adults is ongoing (clinicaltrial.gov NCT05073003) testing the four-component GMMA-based vaccine altSonflex1-2-3, including *S. flexneri* 1b, 2a, and 3a, beyond *S. sonnei* GMMA.

## 5. Conclusions

According to recent estimates, at least 2.5 billion people in LMICs lack access to improved sanitation, and over 884 million lack access to improved drinking water [71]. Global access to improved sanitation and meticulous hand washing represent the ideal solution for preventing transmission of *Shigella*. However, this can be difficult to achieve in all settings, and it can be challenging to sustain, given the financial and logistical constraints in low-resource regions. Vaccination represents the best solution in the short term. However, *Shigella* epidemiology is not static and has been shown to be time- and geography-dependent, complicating vaccine design, already challenged by a limited up to date understanding of serotype burden, need for cross-reactivity of immune responses to different serotypes to simplify vaccine composition, and possible interference among multiple vaccine components. GMMA are highly immunogenic bacterial outer membrane exosomes used as an antigen delivery system [29,30]. Combinations of GMMA, manufactured at high yield and at low cost, seem to be a promising technology for addressing the goal of an affordable vaccine against *Shigella* for LMICs.

Generic methods for cost effective production of GMMA and their formulation have been developed. Starting from a *S. sonnei* monocomponent vaccine, bacteria engineering strategies, manufacturing processes, and analytics have been easily extended to additional serotypes, with the aim of developing an OAg-based multicomponent vaccine with broad protection against the most prevalent serotypes. Furthermore, GMMA have been used as a useful tool for rapidly modifying key features of *Shigella* OAg, investigating the criticality of certain parameters (e.g., OAg of different size or differently decorated with O-acetyl groups) [43,44,45]. GMMA can be directly tested in animal models, allowing questions of scientific interest to be rapidly addressed.

Different OAg-based vaccines are currently in development [69,70,72]. A previous experimental parenteral *S. sonnei* OAg conjugate vaccine showed 71% efficacy against homologous *S. sonnei* infection in children 3 years of age and older but not in the younger children [21]. GMMA represent an innovative and different OAg delivery system. GMMA co-deliver several types of pathogen-associated molecular patterns that confer to GMMA self-adjuvanticity. Further than OAg, GMMA contain *Shigella* proteins that can contribute to efficacy through different mechanisms. *Shigella* strains could be further mutated to express on GMMA additional *Shigella* proteins (e.g., Ipa proteins, virG, absent in current preparations) to increase the breadth and magnitude of the immune response induced, resulting in increased protection against different serotypes. Additionally, heterologous antigens could be presented on GMMA, supporting the development of multicomponent vaccines, covering more pathogens at the same time (e.g., ETEC-*Shigella*) [73].

The *Shigella* GMMA 1790GAHB vaccine was shown in clinical trials to have an acceptable safety profile and to be able to induce a robust immune response in adult humans when administered IM. This demonstrates its feasibility as a vaccine against shigellosis, which at a higher OAg dose is likely to induce sufficient antibodies to protect against moderate to severe diarrhoea due to *Shigella* in areas of the highest need.

A four-valent GMMA-based candidate has just entered a phase 1–2 clinical trial to evaluate its safety and immunogenicity (clinicaltrial.gov NCT05073003). The vaccine will be first administered in European adults and then tested in an age de-escalation trial (from least vulnerable adult population to most vulnerable paediatric population) in a shigellosis-endemic population in Africa. The results of this study will allow the selection of the most appropriate dose for further vaccine development in infants 9 months of age, which is the main target age group for this vaccine.

## Figures and Tables

**Table 1:** Summary of completed clinical studies with GVGH *S. sonnei* 1790GAHB.

Study Phase; (Country) No. of Doses; Type of Study	Study Population (Age)	Test Product(s)—Dose (Route)	No. of Subjects Exposed	Study Status/ Report Status
H03_01TP; (France) 3 doses; Phase 1	Adults (18 to 45 years)	1790GAHB—0.06/1 µg (IM) 1790GAHB—0.3/5 µg (IM) 1790GAHB—1.5/25 µg (IM) 1790GAHB—3/50 µg (IM) 1790GAHB—6/100 µg (IM) Placebo (IM)	8 9 8 8 9 8	Study completed/CSR available in final form
H03_02TP; (UK) 3 doses; Phase 1	Adults (18 to 45 years)	1790GAHB—0.006/0.1 µg (ID) 1790GAHB—0.06/1 µg (ID) 1790GAHB—0.6/10 µg (ID) 1790GAHB—0.3/5 µg (IN) 1790GAHB—1.2/20 µg (IN) 1790GAHB—4.8/80 µg (IN) 1790GAHB—0.3/5 µg (IM) Placebo (ID) Placebo (IN) Placebo (IM)	4 6 6 4 6 6 6 6 7 1	Study completed/CSR available in final form
H03_04TP; (Kenya) 2 doses; Phase 2a	Adults (18 to 45 years)	1790GAHB—1.5/25 µg (IM) 1790GAHB—6/100 µg (IM) Menveo followed by Boostrix (IM)	22 26 24	Study completed/CSR available in final form
H03_01E1TP; (France) 1 dose; Phase 1	Adults (22 to 50 years)	1790GAHB—1.5/25 µg (IM)	35	Study completed/CSR available in final form
H03_03TP (US) 2 doses, Phase 2b	Adults (18 to 50 years)	1790GAHB—1.5/25 µg (IM) Placebo (IM)	36	Study completed/CSR available in final form.

## Data Availability

Not applicable.
